# Atypical, Yet Not Infrequent, Infections with *Neisseria* Species

**DOI:** 10.3390/pathogens9010010

**Published:** 2019-12-20

**Authors:** Maria Victoria Humbert, Myron Christodoulides

**Affiliations:** Molecular Microbiology, School of Clinical and Experimental Sciences, University of Southampton, Faculty of Medicine, Southampton General Hospital, Southampton SO16 6YD, UK; mc4@soton.ac.uk

**Keywords:** *Neisseria* species, *Neisseria meningitidis*, *Neisseria gonorrhoeae*, commensal, pathogenesis, host adaptation

## Abstract

*Neisseria* species are extremely well-adapted to their mammalian hosts and they display unique phenotypes that account for their ability to thrive within niche-specific conditions. The closely related species *N. gonorrhoeae* and *N. meningitidis* are the only two species of the genus recognized as strict human pathogens, causing the sexually transmitted disease gonorrhea and meningitis and sepsis, respectively. Gonococci colonize the mucosal epithelium of the male urethra and female endo/ectocervix, whereas meningococci colonize the mucosal epithelium of the human nasopharynx. The pathophysiological host responses to gonococcal and meningococcal infection are distinct. However, medical evidence dating back to the early 1900s demonstrates that these two species can cross-colonize anatomical niches, with patients often presenting with clinically-indistinguishable infections. The remaining *Neisseria* species are not commonly associated with disease and are considered as commensals within the normal microbiota of the human and animal nasopharynx. Nonetheless, clinical case reports suggest that they can behave as opportunistic pathogens. In this review, we describe the diversity of the genus *Neisseria* in the clinical context and raise the attention of microbiologists and clinicians for more cautious approaches in the diagnosis and treatment of the many pathologies these species may cause.

## 1. Introduction

The genus *Neisseria* is comprised of Gram-negative, *Betaproteobacteria* species belonging to the family *Neisseriaceae*, order *Neisseriales*. To date, about 30 *Neisseria* species have been reported (https://pubmlst.org/bigsdb?db=pubmlst_neisseria_isolates). These species are thought to be restricted to humans generally, although some have been isolated from other mammals or environmental sources [1]. Most of these organisms colonize mucosal surfaces, usually without causing overt pathology, and are therefore regarded as components of the host normal microbiota [2]. However, two species have evolved to cause disease in humans and, as such, are the only two human-restricted pathogens of the genus: *Neisseria gonorrhoeae* and *N. meningitidis* [3]. These two microorganisms are closely related and yet highly adapted to their respective host niches, causing entirely different clinical pathologies [4].

*N. gonorrhoeae* (the gonococcus) is an obligate pathogen that primarily colonizes the mucosal epithelium of the male urethra and female endo/ectocervix, causing the sexually transmitted disease gonorrhea. The gonococcus was discovered by Albert L. Neisser, who in 1879 described the presence of characteristic micrococci in gonorrheal pus from male and female patients [5]. Clinical symptoms for gonococcal genital infection develop as a consequence of neutrophil influx at the sites of mucosal colonization [6]. In men, infection of the urethra causes urethritis and painful discharge, and in women, localized infection of the ectocervix and endocervix leads to a mucopurulent cervicitis. However, clinical symptoms in women are more likely to go unnoticed because neutrophil infiltration does not affect the same niche as urination and pain is often absent. Although ecto/endo-cervicitis in women is commonly asymptomatic, several studies report that asymptomatic infections are indeed common in both genders [6,7,8,9]. In approximately 10–25% of untreated women, gonococci can ascend into the upper reproductive tract (through the endometrium, uterus, Fallopian tubes to ovaries and peritoneum). The host response to this ascending infection can manifest as the clinical syndrome of Pelvic Inflammatory Disease, which can leave patients with long-term and/or permanent sequelae such as chronic pelvic pain, Fallopian tube damage, endometritis, ectopic pregnancy, and infertility [6,10]. These outcomes impact significantly on the health of women worldwide.

Gonococcal infections are mainly localized in the genitourinary tract, but atypical infections can occur at other anatomical sites, as a consequence of Disseminated Gonococcal Infection (DGI), which occurs rarely, or as primary infections due to direct interaction of the pathogen. Treatment of gonorrhea has relied on antibiotics since the first introduction of penicillin in the 1940s, but this and each subsequent antibiotic class introduced has failed to treat gonococcal infections for long, due to the remarkable ability of gonococci to rapidly develop resistance. Worryingly, gonococci resistant to last-resort antibiotics are circulating now and compromising treatment. Thus, the pathogen is on the World Health Organization (WHO) ‘high priority’ list for research into discovering and developing new antimicrobials (https://www.who.int/medicines/publications/WHO-PPL-Short_Summary_25Feb-ET_NM_WHO.pdf). Furthermore, there are no vaccines for gonorrhea. Vaccine development remains a considerable challenge and it is still in “advanced early stage R&D” (https://vaccinesforamr.org/).

The presence of *N. gonorrhoeae* is indicative of infection, as gonococci are not part of the normal microbiota of the urogenital mucosa. Furthermore, colonization without inflammation is not considered commensalism, but an asymptomatic infection instead [3]. However, how far away is the gonococcus from being considered a commensal organism? Commensalism (literally ‘to eat at the same table’) is one form of symbiosis, a biological relationship between two organisms of different species, where one organism benefits while the other is generally unaffected. An organism existing in a commensal state should not elicit a vigorous and sustained host response, since host damage would not provide any selective advantage. *N. gonorrhoeae* has co-evolved with its human host for a long time, which might have resulted in a reduced/modulated pathogenic potential that benefits gonococcal replication and survival and avoids clearance [3,6]. The gonococcus has evolved several mechanisms to enable it to evade recognition and attack from human innate and adaptive immune systems [6,10]. Gonococci can survive and persist in the host using immunosuppressive mechanisms such as binding and inactivating components of the complement cascade [11,12], sialylating its lipo-oligosaccharide (LOS) to hide from the complement system [3,13] and also adapting to changing oxygen and nutrient concentrations [6,14,15]. Furthermore, although asymptomatic infection increases the possibility of complications, it promotes efficient sexual transmission from unaware individuals [6].

*N. meningitidis* (the meningococus) is a commensal of the human nasopharyngeal microbiota that has the potential to become invasive and cause cerebrospinal meningitis and septicemia, with significant mortality and morbidity worldwide [16]. Therefore, it might be more appropriate to describe *N. meningitidis* as an opportunistic pathogen, rather than as a commensal. The *Diplococcus intracellularis meningitidis* was discovered by Anton Weichselbaum in 1887 in the cerebrospinal fluid of patients with ‘epidemic cerebrospinal meningitis’ [17,18] and it was later classified as a member of the genus *Neisseria*. Today, the biology of meningococcal asymptomatic carriage and the genetic basis for the observed virulence of some disease isolates is still a matter of investigation [19]. The clinical symptoms induced by meningococcal infection reflect unrestrained compartmentalized intravascular and intracranial bacterial growth and host inflammation. Systemic (Invasive) Meningococcal Disease (SMD) can be classified into four distinctive disorders: i) Shock without meningitis (fulminant septicemia), ii) shock and meningitis, iii) meningitis without shock, and iv) meningococcemia without shock or meningitis (mild SMD, where patients usually present with fever and may also have a petechial rash) [20]. The most common presentation of SMD is meningitis, whilst fulminant meningococcal septicemia has the highest mortality rate [21]. However, other atypical, but frequent, infections can be manifested, which may sometimes be independent of preceding septicemia and mistaken also for other more common infections associated with different bacterial pathogens [21,22,23,24]. 

Worldwide, there are ~87 million cases of gonorrhea reported annually with the highest burden in low-to-middle income countries [25]. This is probably an underestimate due to unreported asymptomatic infections. By contrast, the cases of SMD have fallen dramatically. Based on data from the recent Global Meningococcal Initiative meeting on preventing meningococcal disease worldwide [26], a crude calculation of recent global case numbers can be made from the case incidence per 100,000 population for countries reporting infections. Globally, the number of cases can be estimated at ~14,000, and this low number is due to the dramatic reduction in cases of serogroup A disease in the ‘meningitis belt’ countries of sub-Saharan Africa. The burden of SMD has always been in the ‘meningitis belt’ and prior to introduction of MenAfriVac, the incidence of SMD cases was ~100/100,000, which equated to ~300,000–600,000 cases annually (depending on population estimates). By contrast to the typical infections of gonorrhea and SMD, the case numbers for atypical infections with *Neisseria* spp. are not known and difficult to estimate in global numbers. Moreover, any estimates of the true burden of these atypical infections are probably underestimates, due to misdiagnoses. Nevertheless, the increased number of published case reports suggests that atypical infections are rising, e.g., in cases of urogenital meningococcal infections, which can be attributed to changing sexual behaviors, notably the increased practice of oral sex has allowed *N. meningitidis* to colonize a new niche (see Section 3.1).

In general, *Neisseria* species are believed to be extremely well adapted to their primary host colonization niches and lacking the plasticity to adapt to alternative niches. It is a reasonable assumption that particular genetic features account for their unique phenotypes, their virulence potential (i.e., the development of accidental versus obligate pathogenicity) and their ability to adapt to their corresponding niche-specific conditions. The molecular bases for these qualities have yet to be wholly elucidated [4,27]. However, isolation of both gonococci and meningococci from sites other than their corresponding natural niches has been reported time and again [28,29,30,31,32]. In addition, infections with commensal *Neisseria* species behaving as opportunistic pathogens have been described, with the oldest reports dating to the beginning of the C20th (extensively reviewed in [33]). In this current review, we provide readers with a broad scenario of ‘atypical’ *Neisseria* infections, with the aim to explore the biological complexity of the genus and raise awareness of these apparently not uncommon events, which may lead to misdiagnosis and consequent inappropriate/ineffective medical treatment. A glossary for the medical terms used throughout this review is provided in Table S1.

## 2. Atypical Infections with *N. gonorrhoeae*

### 2.1. Disseminated Gonococcal Infections (DGIs)

Along with complications from untreated, ascending, female genital tract infections, gonococci can, on rare occasions, enter the bloodstream and cause DGI. Disseminated infection is one of the major threats of gonococcal infection, since the outcome is potentially fatal [34]. Sequelae generally associated with DGI are infectious arthritis, rash, endocarditis or meningitis, resulting mainly from blood dissemination of *N. gonorrhoeae* from primary sexually acquired mucosal infection [35,36]. DGI should also be suspected on appearance of tenosynovitis, polyarthralgia and skin lesions, although these clinical presentations are more commonly associated with gonococcal bacteremia [37].

Neurological manifestations of gonorrhea were observed possibly as early as 1805 by Home [38,39]; however, the first definite case of meningitis attributable to *N. gonorrhoeae* was reported in 1922 [40]. Furthermore, *N. gonorrhoeae* was first implicated as a potential cause of endocarditis by Ricord in 1834 [41,42], but it was not until 1895 that Thayer and Blumer were able to recover this organism from the blood and from lesions on the affected valves of a patient with apparent endocarditis [42,43]. A second case of septicemia with subsequent ulcerative endocarditis due to gonococcal infection was reported in 1899 [44] (Table 1). Despite DGI being a rare complication, its incidence is currently increasing relative to the steady increase in the incidence of gonorrhea worldwide [45]. 

In common with all other *Neisseria* species, gonococci do not have an enhanced ability to leave their normal colonization niches, probably due to their reduced capacity to survive systemically [3]. However, *N. gonorrhoeae* strains associated with DGI are more serum resistant than strains isolated from localized infections [46]. Although *N. gonorrhoeae* lacks a capsule polysaccharide (CPS) to protect itself against serum complement-mediated lysis and opsonophagocytosis, the organism has evolved mechanisms to evade recognition and attack from the human complement system [3,11,12,13]. Certain gonococcal isolates are more disposed than others to become systemic, and it is presumed that both bacterial and host factors contribute to DGI [47,48]. Indeed, a variable genetic island present in *N. gonorrhoeae* and absent in *N. meningitidis* and in all commensal *Neisseria* species, was related to an ability of DGI-associated gonococcal isolates to become systemic [198]. Particular types of this horizontally acquired collection of chromosomally localized genes, i.e., the ones carried preferentially by DGI isolates, confer *N. gonorrhoeae* with a serum resistance locus and encodes also for a peptidoglycan hydrolase that is similar to bacteriophage transglycosylases. Expression of this peptidoglycan hydrolase may correlate with increased peptidoglycan-cytotoxin production [199], thus contributing to enhanced pathogenicity and increased ability of gonococci to survive systemically. Furthermore, all of the different types of this genetic island encode homologues of F factor conjugation proteins, suggesting an involvement in a conjugation-like secretion system, providing DNA for natural transformation [198]. 

### 2.2. Gonococcal Oral and Nasopharyngeal Infections

Gonococcal nasopharyngeal infection could potentially result as a consequence of DGI [55], although it is more generally correlated to preceding orogenital contact [56]. Conversely, disseminated gonorrhea from a primary pharyngeal infection also has been described [57]. The presence of *N. gonorrhoeae* in the human pharynx is reported frequently [28,32,56,58,59,63], probably more so than meningococcal infections of the cervix or the urethra (see below, Section 3.1). Frazer and Menton reported in 1931 a rare case of gonococcal stomatitis and stated that about 40 other cases had been recorded previously since Neisser discovered the gonococcus in 1879, although with no complete proof that the gonococcus was the causative organism [200]. Copping in 1954 [201] and Schmidt et al. in 1961 [202] subsequently reported clinical cases of gonococcal stomatitis, and several other cases with similar clinical presentations have been recorded ever since [203,204]. In 1953, Diefenbach described an infection of the parotid gland with *N. gonorrhoeae* following fellatio of a man with confirmed urethral gonorrhea [64]. Fiumara et al. in 1967 described the first report of gonococcal pharyngitis [60] and two years later, in 1969, Cowan reported a case of a female patient with gonococcal cervicitis and urethritis who developed gonococcal ulceration of the tongue [52]. Today, cases of gonococcal nasopharyngeal infections are reported commonly [205]. The presence of gonococci in the pharynx correlates poorly with symptoms of sore throat [56,59], and cases of symptomatic pharyngitis may be caused by other sexually transmitted agents, particularly in those cases of preceding orogenital contact [28]. However, rare cases of symptomatic gonococcal pharyngitis have been described [61] (Table 1). Interestingly, in the UK in 2016, the first global failure of treating pharyngeal gonorrhea was reported, caused by an eXtensively Drug Resistant (XDR) gonococcus with resistance to both ceftriaxone and azithromycin [206]. Furthermore, in the UK in 2018, a case was reported of a male diagnosed with urethral and pharyngeal gonorrhea; antibiotics cured the urethral infection, but pharyngeal infection was resistant to ceftriaxone, doxycycline, and spectinomycin and finally required intravenous ertapenem for eradication [207]. The increase in pharyngeal gonorrhea is a global concern, enabling both the spread of XDR gonococci and potentially leading to untreatable infections, as drug penetration of the pharynx is poor [205].

### 2.3. Gonococcal Ophthalmia

*N. gonorrhoeae* can colonize the human ocular mucosa as an alternative site of infection. When it occurs in neonates, known as gonococcal *ophthalmia neonatorum*, transmission of *N. gonorrhoeae* and subsequent development of eye infection in the newborn often occurs during delivery and as a direct consequence of exposure to infectious vaginal secretions [66,67]. Vertical transmission of *N. gonorrhoeae* is still possible even with delivery via Caesarean section [68,69,70], which may also cause, although very rarely, some other complications in the neonate apart from ocular infections, such as gonococcal infection of the fetal scalp [73]. Moreover, the above symptoms worsen in cases where gonococcal scalp abscess and necrosis become a focus for disseminated infection [62] (Table 1).

Gonococcal infection of newborn eyes, although frequently mild, can be rapidly destructive and lead to corneal scarring and blindness. In severe cases, corneal ulceration ensues, with probable perforation of the globe and consequential panophthalmitis [62]. Although most cases of gonococcal *ophthalmia neonatorum* are self-limiting and generally benign with appropriate treatment, the infected conjunctivae occasionally serve as a portal of entry for gonococci to induce septicemia, meningitis, arthritis, and/or other manifestations of DGI [49,54] (Table 1).

While typically thought of as a disease in neonates, gonococcal conjunctivitis is an issue also for other age groups. The infection is still reported infrequently in adults and transmission of *N. gonorrhoeae* generally occurs via direct sexual contact with infective secretions [71,72] (Table 1). Indirect transmission, e.g., manually or via fomites, is thought to be less likely, since the microorganism does not survive for long outside its human host. Unlike more common forms of bacterial conjunctivitis in adults, gonococcal infection can cause corneal perforation requiring surgical repair which, if left untreated, could lead to permanent blindness within hours [65,208]. Therefore, rapid arrest of the disease in adults is also essential.

### 2.4. Gonococcal Mastitis

Mastitis is infectious or non-infectious inflammation of the breast, and mastitis caused by *N. gonorrhoeae* infection is extremely rare. Gonococcal mastitis was first reported in the literature in 1993 [74] and only three other similar clinical cases have been described since [75,76,77]. All of the patients in these cases had healed nipple piercings prior to oral-nipple contact, and no other organisms were isolated. *N. gonorrhoeae* cutaneous abscesses in non-genital sites, such as the abdomen, hand, and fetal scalp, have been associated initially with DGI, secondary to disseminated disease [53]. However, other than by hematogenous metastasis from the site of a primary infection, gonococcal abscesses can also occur as a result of direct inoculation or local spread and are often preceded by skin barrier breakdown. This is the case for all four reports of gonococcal mastitis, where the presence of a piercing probably disrupted the skin barrier, predisposing to abscess formation upon exposure to the organism [74,75,76,77] (Table 1).

## 3. Atypical Infections with *N. meningitidis*

### 3.1. Meningococcal Genitourinary Tract Infections

*N. gonorrhoeae* and *Chlamydia trachomatis* are the two most common pathogens colonizing the male and female urogenital tract mucosa [209]. However, *N. meningitidis* can be sporadically pathogenic in the genitourinary tract, as first reported by Murray in 1939 [82]. In several subsequent reports, the presence of *N. meningitidis* in the urethra was not associated with genital symptoms [78,83,84,95]. However, genital infections caused by meningococci may sometimes present similar clinical symptoms to classical gonorrhea, e.g., purulent penile discharge and urethritis, and cervicitis/vaginitis [29,30,85,86,87,93,94] (Table 1). 

A recent analysis of urogenital and rectal infections revealed co-colonization with encapsulated, hyperinvasive meningococci and closely related MultiDrug-Resistant (MDR) gonococci [88]. The main concern with co-infection is an increased chance that meningococci acquire gonococcal antimicrobial resistance genes. Co-existence of meningococci with gonococci poses a clear risk to public health, as the emergence of menincococcal strains with expanded antimicrobial resistance could contribute to therapeutic complications in the treatment of meningococcal disease. In fact, urogenital meningococcal isolates possessing gonococcal plasmids have been described [89]. Furthermore, expansion of a US non-groupable (unencapsulated) urethritis-associated *N. meningitidis* clade (NmNG) with concurrent acquisition of *N. gonorrhoeae* alleles has been reported recently [90,91]. However, acquisition of common gonococcal antimicrobial resistance factors by this clade has not been described to date. Nonetheless, in the study from Retchless et al. [90], the authors suggested that since the clinical presentation of meningococcal urethritis mirrors that of gonococcal infections, ‘the evolutionary forces that resulted in high rates of antimicrobial resistance among *N. gonorrhoeae* may lead to the same result among these *N. meningitidis*’. 

The reasons why a commensal organism of the human nasopharynx may become pathogenic and the molecular mechanisms that perturb the host-bacterium equilibrium are mostly unknown. A whole-genome comparison of disease and carriage meningococcal strains provided insights into the virulence evolution of *N. meningitidis* and it suggested that this bacterium emerged as an encapsulated human commensal from a common ancestor with *N. gonorrhoeae* and *N. lactamica*, subsequently acquiring the genes responsible for capsule synthesis via horizontal gene transfer [16]. The *cps* locus required for capsule synthesis consists of several regions, some of which might belong to the *Neisseria* core genome because they can be found in many other *Neisseria* spp. However, the regions containing the genes required for capsule synthesis, modification, and transport can be found only in the encapsulated meningococcal strains [16]. Some of these genes are highly similar in sequence and operon organization to homologous genes in the *Pasteurella multocida* genome [16]. These observations are in line with previous studies reporting horizontal gene transfer from encapsulated *Haemophilus influenzae* (a member of the *Pasteurellaceae* and a resident of the human airways) to *N. meningitidis* [210]. Thus, horizontal gene transfer between different bacterial species present in the oro-nasopharyngeal microbiota may drive evolutionary events.

Expression of capsule is the only feature that has been linked convincingly to the pathogenic potential of *N. meningitidis*: capsule mediates protection from desiccation during transmission and mediates resistance against complement-mediated lysis and opsonophagocytosis during SMD [211,212,213,214,215,216]. However, although meningococcal carriage isolates are frequently unencapsulated due to absence of the genetic island encoding for capsule synthesis [217], carriage isolates expressing capsule otherwise associated with disease have been reported [218,219,220]. Therefore, the conclusion that the capsule is necessary, but not sufficient, to confer virulence would seem to be fair, except for those unique cases of meningococcal urethral infections with unencapsulated isolates belonging to the US NmNG clade described above [91]. Since capsule expression contributes to virulence during SMD [211], disruption of the *cps* locus in the US NmNG urethritis-associated clade was expected to limit the risk of SMD from this clade. However, five unencapsulated isolates from SMD cases were identified, and primary urethral colonization was proposed to contribute to subsequent sepsis caused by this NmNG clade [90]. These urethritis-associated isolates have adapted particularly to the urogenital environment with two unique molecular fingerprints: A multi-gene deletion at the capsule synthesis locus that enhances mucosal adherence, and acquisition of the gonococcal denitrification pathway by gene conversion that promotes anaerobic growth [92]. These phenotypic changes, and potentially others, suggest that multiple independent evolutionary events have selected this newly emergent lineage meningococcal clade to better assimilate into the same niche first populated by gonococci, and thus become a successful urogenital pathogen [90,92], but one that maintains its competence to cause SMD [90]. 

In previous studies, the route by which *N. meningitidis* reached the genital tract was highly speculative. For example, in Murray’s report, the isolation of meningococci from the urogenital tract of male patients was associated with meningococcal septicemia in which the testes and epididymides were involved [82]. In two female patients described by Keys et al. [95], the presence of endocervical meningococci was also associated with meningococcemia, but it was unclear whether meningococcemia preceded cervical infection, or vice versa. In the majority of other cases described in the literature, however, patients from whom meningococci were isolated from the cervix or the urethra, did not present any signs of septicemia, and it seems unlikely that the organism reached the genital tract by the hematogenous route. In the majority of these cases, transmission by orogenital sexual activity seems probable [28,29,78,84,85]. Several cases of neonatal meningococcal meningitis associated with maternal cervical-vaginal colonization have also been reported [79,80,99], with the first report in 1997 by Harriau et al. of associated oropharyngeal colonization of the male partner [96]. In this study, the phenotypic and genomic identities of meningococcal strains isolated from both the endocervix of the infected pregnant woman and her male partner was the first clear evidence for *N. meningitidis* cross-colonization between sexual partners. In addition, the possibility of self-transmission from the pharynx to the urethra via the hands also certainly exists, as suggested by a case report of a male heterosexual patient who harbored organisms of the same serotype and sensitivity patterns in both sites [28] (Table 1).

### 3.2. Meningococcal Ophthalmia

By contrast to gonococcal infections of the eye [6], meningococcal eye infections are more rare. Since *N. gonorrhoeae* and *N. meningitidis* cannot be differentiated with Gram’s stain, because they both appear as Gram-negative diplococci [221], clinical symptoms of apparent gonococcal ocular infections should be approached with caution so as not to misdiagnose the odd cases of meningococcal *ophthalmia*, which may develop further into more severe sequelae. Meningococcal conjunctivitis is a rare condition that can have devastating ocular and systemic complications, and hence topical antibiotics alone are insufficient for treatment [100,101]. Simple conjunctivitis can progress into endophthalmitis, which is accompanied usually by severe pain, loss of vision, and redness of the conjunctiva and the underlying episclera. Meningococcal endophthalmitis presents variably with sepsis [102,112,113,121], meningitis [114,115], or isolated ocular symptoms without systemic illness [112,116,117,118,119], although subsequent development of other expressions of meningococcal disease should not be ruled out [103,104,105]. Thus, delayed or incorrect treatment of meningococcal ocular infections ultimately risks blindness, disability, or death [120] (Table 1).

Natural populations of *N. meningitidis* carried in the nasopharynx are not associated with invasive disease [217], and yet retain the potential to become pathogenic by entering the bloodstream, crossing the blood–cerebrospinal fluid barrier (BCSFB) and invading the meninges [222]. Invasion of the BCSFB and blood–ocular barriers by meningococci suggests common antigenic expression in meningeal and ocular microvascular endothelial beds. The possibility of meningococci reaching the ocular site by the hematogenous route is feasible but unproven [103]. Indeed, meningococcal ocular infections are most commonly associated with preceding SMD and rarely occur in isolation. Nonetheless, cases of primary meningococcal conjunctivitis (with no associated symptoms of SMD) resulting from close contact with another patient diagnosed with meningitis [101] and even through transmission from direct ocular contact with saliva from apparently healthy individuals [106,107], suggest that the routes of transmission to the eye may differ in each particular clinical case (Table 1).

Unusual cases of neonatal meningococcal conjunctivitis have also been reported. The first report of primary neonatal meningococcal conjunctivitis is from Hansman and dates back to 1972 [108]. In this study, the source of infection was not established, since cultures of cervical and urethral swabs collected from the mother failed to yield *Neisseria* (Table 1). Hansman therefore considered that the neonatal infection probably originated by contact with a different meningococcal carrier, possibly a member of the hospital staff. Subsequently, other cases of primary meningococcal conjunctivitis in newborn infants acquired by direct contact with an exogenous meningococcal source have been described [109]. More recently, an unusual case of vertical transmission of *N. meningitidis* to a neonate acquired at delivery, with subsequent development of neonatal primary meningococcal conjunctivitis, was reported by Fiorito et al. [97]. In this report, the source of transmission to the neonate was confirmed to be the mother’s endocervical infection (see above, Section 3.1), and sexual cross-transmission of the same strain with her partner was also proved [97]. This case study from Fiorito et al. is the first report of an alternative transmission pathway by which *N. meningitidis* may reach and colonize the eye that is different to transmission via the hematogenous route and/or via direct contact with an exogenous source [97,110]. Meningococcal neonatal purulent conjunctivitis and consequential sepsis associated with asymptomatic carriage of *N. meningitidis* in the mother’s vagina and both parents’ nasopharynx has also been described [81]. In this study, it is possible that the bacteria in the newborn were acquired by vertical transmission from the mother’s vagina during delivery, and the presence of bacteria in the nasopharynx of both parents suggested also horizontal transmission amongst them [81] (Table 1). 

Neonatal meningococcal meningitis following meningococcal conjunctivitis, where the eye may have been the portal of entry after intrapartum contamination with the pathogen, is rare [223]. In the cases reported by Sunderland et al. in 1972 [80] and Jones et al. in 1976 [79], the ultimate outcome of disease was child death. The first report of a surviving newborn infected in the same manner was published by Ellis et al. in 1984 [111] (Table 1). Thus, quick and precise diagnosis and treatment of meningococcal conjunctivitis in neonates is crucial, as inappropriate management of a primary eye infection with *N. meningitidis* may have severe implications for the newborn’s health.

## 4. Infections with Commensal *Neisseria* Species

Non-pathogenic *Neisseria* species comprise part of the commensal bacterial microbiota of the human and animal oropharynx, but might occasionally behave as opportunistic pathogens [1,224]. Whether this commensal population contributes to human health and/or impacts on colonization and disease caused by bacterial pathogens remains to be elucidated. Kim et al. (2019) [225] reported the first clear evidence that commensal *Neisseria* can kill *N. gonorrhoeae* through a DNA-mediated mechanism based on genetic competence and DNA methylation state, accelerating clearance of gonococci in a DNA-uptake-dependent manner. Consistent with these findings, the authors suggested that the antagonistic behavior of commensal *Neisseria* toward their pathogenic relatives may negatively affect *N. gonorrhoeae* colonization and that DNA is a potential microbicidal agent against drug-resistant gonococci [225].

There is ample evidence in the literature, however, that these ‘apparently harmless’ inhabitants of the oropharynx are capable of producing infection in a wide variety of anatomical sites including the heart, nervous system (meningitis), bloodstream (septicemia), respiratory tract, bone marrow, skin and possibly the genital tract. Many of these infections occur possibly secondary to a primary infection elsewhere, e.g., subsequent invasion of the bloodstream by *Neisseria* from the oropharynx may lead to endocarditis and meningitis, with an overlap of the clinical features of these conditions [1,33].

### 4.1. Endocarditis

To our knowledge, the first recorded case of endocarditis caused by a ‘presumably’ commensal *Neisseria* species was probably from Coulter in 1915 [226], although the organism, referred to as a ‘Gram-negative *Micrococcus*’, was inadequately characterized. Regardless, Coulter’s study was considered by Johnson in his literature review in 1983 on the pathogenic potential of commensal *Neisseria* species [33]. Schultz described the first confirmed case of endocarditis as a consequence of infection with a commensal species of *Neisseria* in 1918, identified as ‘*Micrococcus pharyngitidis-siccae*’ (*N. sicca*, as we know it today) [173]. Graef et al. described a case of endocarditis caused by this same organism in 1932, but referred to it as ‘*Micrococcus pharyngis siccus*’ [174]. Since then, many other cases of confirmed endocarditis caused by *N. sicca* have been recorded [175,176]. Other commensal *Neisseria* species have also been associated with heart infections, e.g., *N. bacilliformis* [122,123], *N. elongata* [135,136], *N. flava* [139,140], *N. flavescens* [141,142], *N. mucosa* [157,158,159], *N. perflava* [170,171], and *N. subflava* [183,184], (Table 1).

### 4.2. Meningitis and Septicemia

In 1908, Wilson described a case of cerebrospinal meningitis caused by *Micrococcus catarrhalis* (also previously described as *N. catarrhalis*, now *Moraxella (Branhamella) catarrhalis*) [227]. Since then, *Neisseria* spp. other than *N. meningitidis* and *N. gonorrhoeae* identified as causing meningitis include *N. flavescens* [143,144], *N. lactamica* [149,150], *N. mucosa* [160,161], *N. sicca* [177,178], and *N. subflava* [185,186,187]. Moreover, several non-gonococcal, non-meningococcal *Neisseria* species have been isolated from blood cultures, many of which have been associated with infections including endocarditis (see above, Section 4.1), septicemia and meningitis [124,127,128,137,139,145,146,151,162,169,186,190] (Table 1).

### 4.3. Respiratory Tract Infections

The association of *Neisseria* spp. with respiratory tract infection pathologies is challenging as *Neisseria* organisms, with the sole exception of the gonococcus, are known to inhabit harmlessly the upper respiratory tract [2]. Nevertheless, there is increasing evidence to suggest that *N. catarrhalis* (*M. catarrhalis*), can cause infections in the upper and lower respiratory tract, with associated symptoms of otitis, laryngitis, bronchitis, bronchiectasis, pneumonia, or sinusitis [228,229,230,231,232,233,234,235,236,237,238]. Similarly, *N. bacilliformis* [124], *N. canis* [125], *N. flavescens* [147], *N. lactamica* [152,153,154], *N. mucosa* [163], *N. sicca* [179], and *N. weaveri* [191], have also been reported to cause respiratory tract infections (Table 1).

### 4.4. Genitourinary Tract Infections

Isolation of Gram-negative diplococci from genital tract smears is generally thought to be evidence of gonococcal infection [6]. However, as discussed in Section 3.1, meningococcal genitourinary tract infections do also occur and similarly, several commensal *Neisseria* spp. have been isolated from the genitourinary tract, although it is not clear whether these organisms cause any pathological changes or symptoms when colonizing this anatomical site. Nevertheless, absence of symptomatic disease does not necessarily imply that these other *Neisseria* spp. do not have pathogenic potential, since infection with the gonococcus is frequently asymptomatic, especially in women [239]. The earliest reports of non-gonococcal, commensal *Neisseria* spp. present in the genital tract include *N. catarrhalis* (*M. catarrhalis*) [148,180,240,241,242], *N. flavescens* [148], *N. lactamica* [155,156], *N. sicca* [148,180,181], and *N. subflava* [148,180,188]. More recently, further examples of male genitourinary infections with *N. cinerea*, *N. lactamica*, and *N. mucosa* have been described [129] (Table 1).

### 4.5. Other Infections, Epidemiology, and Factors Possibly Influencing Disease Development

Since the early 1900s, numerous clinical cases have been described in the literature of commensal *Neisseria* spp. capable of colonizing a wide variety of anatomical sites other that the nasopharynx and causing disease. Thus, only exemplar reports are cited in this current review (Table 1). These cases and other pathologies associated with infection with non-pathogenic *Neisseria* spp., such us peritonitis [131,192], purulent wound and cellulitis [126], osteomyelitis [138], skin ulceration [195], visceral botryomycosis [165], neonatal conjunctivitis [132,133], and cystitis [168] have been thoroughly reviewed by Liu et al. in 2015 [1] (Table 1).

From an epidemiological perspective, infections with commensal *Neisseria* spp. occur as singular events rather than as outbreaks, except for probably one single event of epidemic meningitis caused by *N. flavescens* reported in 1930 [143]. This epidemiology suggests minimal person-to-person transmission, and development of the disease may probably be due to endogenous spread of the organism from a primary infected site (oropharynx). In this scenario, a host prone to infection (e.g., immunocompromised) and/or enhanced virulence of the particular infective strain may determine the outcome of disease, as in cases of DGI [47,48] and SMD [243]. For instance, access of the organism to the bloodstream as a direct consequence of a preceding oral trauma, such as in cases of endocarditis, meningitis and septicemia, suggests that the infective organisms should be resistant to the bactericidal activity of normal human serum. Whether ‘commensal’ *Neisseria* isolated from blood are serum resistant in comparison to isolates of the same species confined to the nasopharynx, remains to be elucidated. Alternatively, host immuno-deficiency may predispose susceptible individuals to systemic infections, as observed with patients suffering from DGI and SMD [244]. Furthermore, successful colonization of the anatomical site, which requires organism attachment to host cells to establish commensalism, precedes blood invasion and intravascular survival. Once established, the organism should be capable of resisting clearance by host immune defenses, perhaps through molecular mechanisms similarly described for the gonococcus [6,10,245,246]?

## 5. Antimicrobial Treatment of Typical and Atypical *Neisseria* Infections

Complicated gonorrhea (DGI) and meningococcal disease are both life-threatening infections that, even after initiation of appropriate treatment, may progress rapidly and be potentially fatal. Timely diagnosis is key for effective management and both are crucial to prevent or reduce the complications of infection. Thus, increased awareness is needed for i) the possibility of atypical infections with commensal *Neisseria* spp. resembling those clinical symptoms associated with *N. gonorrhoeae* and *N. meningitidis* infections, ii) the likelihood of atypical infections with these pathogens in alternative anatomical sites, and iii) knowledge on how to treat them effectively. Typical, uncomplicated gonorrhea is usually treated empirically with a short course of antibiotics, without testing for antimicrobial susceptibility. The Centers for Disease Control and Prevention (CDC) recommends a single dose of 250 mg of intramuscular ceftriaxone and 1 g of oral azithromycin (https://www.cdc.gov/std/tg2015/gonorrhea.htm). In the UK, given the rise in resistance to azithromycin, the 2019 guidelines from the British Association for Sexual Health and HIV (BASHH), recommends ceftriaxone 1 g intramuscularly as a single dose (https://www.bashhguidelines.org/current-guidelines/urethritis-and-cervicitis/gonorrhoea-2019/). For DGI, the CDC recommends a variety of antibiotics including ceftriaxone, azithromycin and cefotaxime, depending on the clinical presentation, e.g., arthritis and meningitis. Cefotaxime, ceftriaxone and benzylpenicillin are preferred as initial therapy in patients with a clinical diagnosis of SMD, although alternative antibiotic therapies to treat typical meningococcal disease are also available [247]. In general, similar antimicrobial treatments for atypical infections with *N. gonorrhoeae* and *N. meningitidis* have also proved successful (Table S2). However, antimicrobial prescription for atypical infections with commensal *Neisseria* spp. varies widely depending on the species and on the anatomical site of infection (Table S2). Therefore, precise diagnosis is essential.

## 6. Discussion

**Figure 1:**
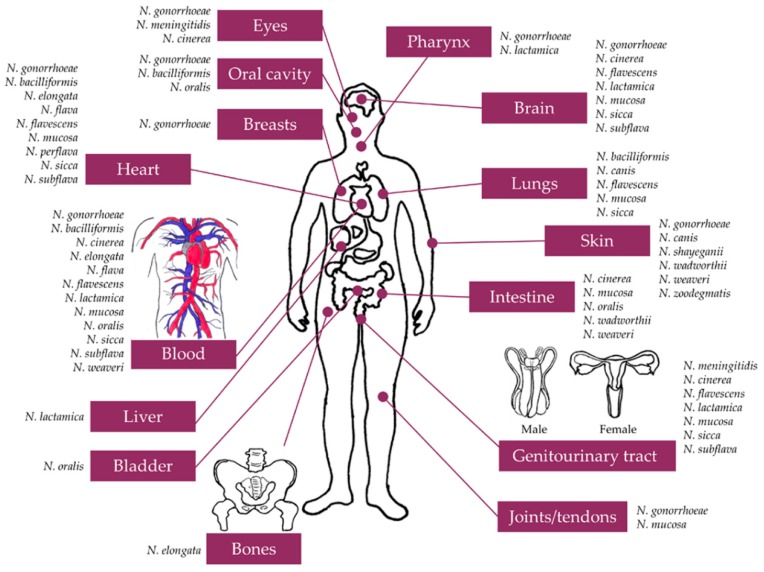
Legend. Only exemplar atypical anatomical sites infected by pathogenic and commensal *Neisseria* species are depicted. Corresponding references for these and other clinical case reports of unusual infections with *Neisseria* species are listed in Table 1. Characteristic (typical) infections with gonococcus (gonorrhea) and meningococcus (meningitis and septicemia) are not included. Many of the unusual gonococcal infections are either associated with preceding DGI or considered the cause of subsequent gonococcal septicemia and/or other manifestations of DGI. Some clinical cases of unusual meningococcal infections are either associated with preceding meningococcaemia or further develop sepsis (SMD) as a consequence of the corresponding primary infection (refer to the main text for more details).

*Neisseria* spp. are highly adapted to the environmental conditions of the unique niches that they colonize. However, the genus *Neisseria* is far more diverse and complex than acknowledged previously. For example, ‘commensal’ *Neisseria* spp. have generally been regarded as harmless organisms of little clinical importance, but it is clear that they can occasionally disseminate from their commensal niche and occupy, survive and proliferate in other anatomical niches and cause serious infections (Figure 1) [1,33]. Conversely, the closely related pathogens of the genus, *N. gonorrhoeae* and *N. meningitidis*, have adapted evolutionarily to their specific niche and cause diseases with distinctive profiles. However, their differences can sometimes be compensated by their biological similarities, which may probably explain those cases in which these two organisms behave in clinically-indistinguishable fashion (Figure 1) [98,248].

Successful colonization of the mammalian host by *Neisseria* spp. requires an initial adhesive interaction between the bacterium and the host mucosal epithelial cell. *Neisseria* adhesion to the exposed epithelia depends on a repertoire of diverse molecules within the bacterial outer membrane (OM) and extending from the bacterial surface and their interplay with specific host cell receptors [249,250,251,252]. Models of *Neisseria* spp. colonization suggest that after initial adhesion, maintenance of association involves bacterial aggregation, microcolony, and biofilm formation and the activation of mechanisms to avoid host immunity [253,254]. Despite the fact that *Neisseria* spp. colonize specific, distinctive niches, ample evidence of these species adhering to and colonizing other anatomical sites, some of which are colonized by more than one species, suggests that pathogenic and commensal *Neisseria* might share conserved surface molecules important for bacterial-host cell interactions. In fact, for classical *Neisseria* infections, a great deal is known about the biology, structure and function of *Neisseria* adhesins, the putative target human cell receptors, the molecular bases of their interactions and the resulting modulation of both *Neisseria* spp. and host cells in response to these interactions. Several excellent reviews cover these topics comprehensively [249,255,256,257,258,259]; nevertheless, we provide the reader with a brief, general discussion on conserved adhesins and other surface molecules important for initial adhesion and colonization, which may possibly help to interpret, from the view of microbiology, the extensive medical records reporting atypical infections with *Neisseria* species. In the case of atypical presentations of *Neisseria* infections in different anatomical sites, specific host cell/receptor–pathogen interactions have not been characterized; thus, an explanation for why and potentially how they occur from the view of host cell biology is still a matter of investigation.

The Type IV pilus is probably the most extensively studied *Neisseria* adhesin. Extending out from the *Neisseria* OM, pili impart twitching motility by rapid extension and retraction, facilitate uptake of foreign DNA to increase transformation frequency and are important for virulence [260]. Meningococci produce two structurally distinct types of pili, Class I and Class II. Gonococci only produce Class I pili, and both gonococcal and meningococcal Class I pili are recognized by murine monoclonal antibody SM1 [261]. Expression of pili in commensal *Neisseria* species has not been characterized as extensively as within *N. gonorrhoeae* and *N. meningitidis*, but a comparative analysis of the pilin gene in pathogenic and non-pathogenic *Neisseria* spp. demonstrated two distinct structural groups—i) the gonococcal and meningococcal Class I pilin-encoding genes and ii) the *N. lactamica*, *N. cinerea* and meningococcal Class II pilin-encoding genes [262]. Expression of pili by commensal and pathogenic *Neisseria* spp. is necessary for primary colonization of the nasopharyngeal and genitourinary niches. Pili also plays a critical role in enabling adhesive interactions of the *Neisseriae* with other anatomical niches and thus occasioning different pathologies. 

The most abundant adhesion/invasion molecules embedded within the *Neisseria* OM are the Opacity-associated (Opa) and Opc proteins. The Opc protein is expressed only in *N. meningitidis* [263]. Although an *opc* pseudogene is present in *N. gonorrhoeae* and some commensal strains of *N. polysaccharea*, significant difference was observed within the region encoding the most surface-exposed loops and there is no evidence of Opc protein expression by these organisms [263,264]. However, Opa protein is abundantly expressed and regulated in gonococci [265], meningococci [266], and the commensal strains *N. subflava*, *N. mucosa*, *N. sicca*, *N. flava*, and *N. lactamica* [267]. Other OM adhesins include the Adhesion and penetration protein (App), the Neisserial Adhesin A protein (NadA) and the *Neisseria hia/hsf* homologue NhhA protein. App is highly conserved across all *Neisseria* species, and the meningococcal App protein amino acid sequence shares ~95% and 73% identity with *N. gonorrhoeae* and *N. lactamica*, respectively [257,268,269]. The extensively characterized Trimeric Autotransporter NadA is present in ~50% of meningococcal strains but absent in *N. gonorrhoeae* and *N. lactamica* [270] and NhhA protein was reported to be expressed in *N. meningitidis* and *N. lactamica*, but not in *N. gonorrhoeae* [271].

Several other surface structures can influence bacterial attachment, e.g., CPS, LOS, and OM porin (Por) proteins. CPS expression by the meningococcus is important for virulence and capsulated and piliated meningococci are cultured from patients with sepsis and meningitis. However, CPS expression is not the only trait essential for the pathogenic potential of *N. meningitidis*. This is demonstrated by the presence of meningococcal carriage isolates expressing CPS that are not associated with disease [218,219,220] and by unique cases of meningococcal urethral infections with unencapsulated isolates [91] (see above, Section 3.1). Furthermore, gonococci and commensal *Neisseria* species do not express CPS and are still capable of causing infections. Similarly, there is high genetic diversity in the *ltg* loci related to the biosynthesis of LOS in pathogenic *Neisseria* and some of these genes are also found in strains considered to be non-pathogenic, e.g., *N. lactamica*, *N. subflava*, and *N. sicca*. However, *ltg* is not carried by all commensal strains [272,273]. Porins comprise up to 60% of the proteins present in the *Neisseria* OM. While most *Neisseria* species express only one Por, meningococci express two, PorA and PorB. The gonococcus is the only other *Neisseria* species known to have a *porA* pseudogene, which is silent due to frameshift and promoter mutations [274]. Phylogenetic analyses suggested an important role for horizontal genetic exchange in the emergence of different porin classes and confirmed the close evolutionary relationships of the porins from *N. meningitidis*, *N. gonorrhoeae*, *N. lactamica*, and *N. polysaccharea* [275]. 

The evolution of specific *Neisseria* adhesins that enable primary colonization and subsequent maintenance of a commensal carriage or progress of disease is in many respects driven by the compliant host [257]. In addition, while it might be true to state that commensal organisms and pathogens share similar adhesins, commensal *Neisseria* may not normally express the profile of virulence-associated proteins required for infection. Yet, the genetic propensity of commensal *Neisseria* species to cause disease does exist and it is reported occasionally (Table 1).

Comparative genomics of commensal human *Neisseria* species revealed that these organisms share a large repertoire of virulence-associated alleles with gonococci and meningococci, probably as a consequence of widespread virulence gene exchange amongst them [257,276,277]. A recent genome-wide analysis by Lu et al. (2019) [4] compared the genomes of 15 *N. gonorrhoeae*, 75 *N. meningitidis* and 7 commensal *Neisseria* spp. (i.e., three *N. lactamica* strains and single examples of *N. mucosa*, *N. weaveri*, *N. zoodegmatis,* and *N. elongata*) to identify genes associated with pathogenicity and niche adaptation. In this study, a core-pangenome analysis found that 452, 78, and 319 gene families were unique to gonococci, meningococci and were shared, respectively. Furthermore, abundant Simple Sequence Repeats, the molecular basis for gene phase variation, was found within these gene sets and were therefore regarded as candidates that related to their pathogenicity and ability to adapt to variable host environments [278,279]. Functional annotation analysis partly verified the relationships among them, but no certain functional information was found for at least one-third of the genes for each gene set [4]. 

Protein–protein interaction analysis (PPI) of unique gonococcal and meningococcal proteins found at least five and four PPI clusters in *N. gonorrhoeae* and *N. meningitidis*, respectively. These were associated mainly with basic substance transport and metabolism, genetic information processing (e.g., replication, transcription and translation), cellular processes (e.g., cell wall/membrane/envelope biogenesis and cell motility), bacteria-environment interactions (e.g., signal transduction, extracellular structures and defense mechanism), nitric oxide metabolic pathways, heme utilization and adhesion systems [4]. These proteins unique to the pathogenic *Neisseria* spp. may well be vital for their pathogenic potential and niche adaptation. Within these clusters, numerous other proteins with unknown function were also detected in the PPI analysis maps and should be investigated further for other possible interactions relevant to the pathogenicity of these species.

In this same study, commensal *Neisseria* strains showed conservation of 14 gene families and shared 39 gene families with gonococci and 11 gene families with meningococci. Interestingly, Lu et al. [4] also reported 1111 gene families that were conserved across all pathogenic and non-pathogenic *Neisseria* spp. These specific and shared genetic features could underlie the apparent differences of niche specialization and the pathogenic potential of meningococci and gonococci. They may lead us also to infer the molecular relationships between phenotypes of the ‘atypical’ infections with both pathogenic and ‘commensal’ *Neisseria* spp. Furthermore, but beyond the scope of this review, it would be worth studying the genomes of isolates from different anatomical sites, which could be partly achieved from analyzing the pubMLST.org/*Neisseria* database (Table S3). This would enable us to compare similarities between different *Neisseria* species causing the same atypical infection and the differences between the same *Neisseria* species with distinct virulence profile(s) (i.e., isolated from different anatomical sites).

## 7. Conclusions

In this review, we highlight the atypical infections that can be caused by pathogenic and commensal *Neisseria* spp., thereby demonstrating how effectively these organisms can colonize different anatomical niches. An increased awareness of this propensity for colonizing multiple sites would suggest a more cautious approach to diagnosing the clinical syndromes normally attributed to infection with the gonococcus or the meningococcus, and guard against dismissing as normal microbiota other *Neisseria* spp. isolated from sites other than the nasopharynx.

## Figures and Tables

**Table 1:** Examples of reported clinical cases of unusual infections with *Neisseria* species.

*Neisseria* species	Anatomical Site of Infection	Disease	Case Report
Pathogenic *Neisseria* species			
*N. gonorrhoeae* **^1^**	Blood	DGI/septicemia	[34,43,44,46,47,48,49]
	Joints	DGI/arthritis	[35,37]
	Heart	DGI/endocarditis	[42,43,44,45,50]
	Skin (extragenital)	DGI/cutaneous infection	[51,52,53]
	Brain	DGI/meningitis	[38,39,40,54]
	Pharynx	DGI/pharyngitis	[55]
		Oro- and nasopharyngeal infections	[32,56,57,58,59,60,61,62]
		Tonsillitis	[63]
	Mouth/lips	Stomatitis	
	Parotid glands	Parotitis	[64]
	Tendon	DGI/tenosynovitis	[61]
	Eye	Keratoconjunctivitis	[31,65]
		Conjunctivitis/*ophthalmia neonatorum*	[49,62,66,67,68,69,70,71,72]
	Scalp	Scalp abscess	[73]
	Breast	Mastitis/breast abscess	[74,75,76,77]
*N. meningitidis* **^2^**	Genitourinary tract	Vaginitis	[29,78,79,80,81]
		Urethritis	[30,82,83,84,85,86,87,88,89,90,91,92,93,94]
		Cervicitis	[78,79,83,85,86,89,90,93,95,96,97,98]
		Anal canal infection/proctitis	[83,86,88,89,90]
		Intrauterine infection	[99]
	Eye	Conjunctivitis	[81,97,100,101,102,103,104,105,106,107,108,109,110,111]
		Endophthalmitis	[112,113,114,115,116,117,118,119,120]
		Panophthalmitis	[121]
Commensal *Neisseria* species **^3^**			
*N. bacilliformis*	Heart	Endocarditis	[122,123]
	Oral cavity/fistula	Submandibular wound	[124]
	Sputum	Possible bronchitis	[124]
	Sputa	Possible bronchitis	[124]
	Lung	Lung abscess	[124]
	Blood	(Insufficient clinical data)	[124]
*N. canis*	Lung	Bronchiectasis	[125]
	Skin	Purulent wound/cellulitis	[126]
*N. cinerea*	Blood	Septicemia	[127,128]
	Brain	Meningitis	[128]
	Genitourinary tract	Genital infections	[129]
		Urinary infection	[130]
	Peritoneum	Peritonitis	[131]
	Eye	Conjunctivitis/*ophthalmia neonatorum*	[132,133]
*N. dumasiana*	Sputum	(Insufficient clinical data)	[134]
*N. elongata*	Heart	Endocarditis	[135,136]
	Blood	Septicemia	[137]
	Bone	Osteomyelitis	[138]
*N. flava*	Heart	Rheumatic heart disease/ventricular septaldefect/endocarditis	[139]
		Endocarditis	[140]
	Blood	Sepsis/conjunctival petechia	[139]
*N. flavescens*	Heart	Endocarditis	[141,142]
	Brain	Meningitis	[143,144]
	Blood	Septicemia	[145,146]
	Lung	Pneumonia/empyema	[147]
	Genitourinary tract	Genital infections	[148]
*N. lactamica*	Brain	Meningitis	[149,150]
	Blood	Septicemia	[145,151]
	Pharynx	Pharyngitis	[152]
	Lung	Cavitary lesion	[153]
		Pneumonia	[154]
	Genitourinary tract	Genital infections	[129,155,156]
*N. mucosa*	Heart	Endocarditis	[157,158,159]
	Brain	Meningitis	[160,161]
	Blood	Septicemia	[145,162]
	Lung	Empyema	[163]
	Genitourinary tract	Genital infections	[129]
		Urinary infection	[164]
	Viscera	Botryomycosis	[165]
	Joints	Arthritis	[166,167]
*N. oralis*	Bladder	Cystitis	[168]
	Gingiva	Healthy gingival plaque/subgingival oral biofilm	[169]
	Blood	(Insufficient clinical data)	[169]
	Urinary tract	(Insufficient clinical data)	[169]
	Paracentesis fluid	(Insufficient clinical data)	[169]
*N. perflava*	Heart	Endocarditis	[170,171]
*N. shayeganii*	Sputum	(Insufficient clinical data)	[172]
	Skin	Arm wound	[172]
*N. sicca*	Heart	Endocarditis	[173,174,175,176]
	Brain	Meningitis	[177,178]
	Blood	Septicemia	[145]
	Lung	Pneumonia	[179]
	Genitourinary tract	Genital infections	[148,180,181]
		Urinary infection	[182]
*N. subflava*	Heart	Endocarditis	[183,184]
	Brain	Meningitis	[185,186,187]
	Blood	Septicemia	[145,186]
	Genitourinary tract	Genital infections	[148,180,188]
		Urinary infection	[189]
*N. wadsworthii*	Skin	Hand wound	[172]
	Peritoneal fluid	(Insufficient clinical data)	[172]
*N. weaveri*	Blood	Septicemia	[190]
	Sputum	Bronchiectasis	[191]
	Peritoneum	Peritonitis	[192]
	Skin	Wound	[193,194]
*N. zoodegmatis*	Skin	Ulceration	[195]

Table 1 Legend. Only exemplar clinical case reports of unusual infections with pathogenic and commensal *Neisseria* species are listed in the Table 1; characteristic (typical) infections with gonococcus (gonorrhea) and meningococcus (meningitis and septicemia) are not included. **^1^** Many of the unusual gonococcal infections are associated with preceding disseminated gonococcal infection (DGI) (consequential of initial gonorrhea) or serve as a portal of entry for gonococcal septicemia and/or other manifestations of DGI. **^2^** Some clinical cases of unusual meningococcal infections are either associated with preceding meningococcemia or further develop sepsis (systemic (invasive) meningococcal disease (SMD)) as a consequence of the corresponding primary infection. **^3^** Commensal *Neisseria* species are not associated with disease, although they may behave as opportunistic pathogens. In many of these cases, an overlap of clinical features for different conditions is generally observed (e.g., invasion of the bloodstream by *Neisseria* may also occur in cases of endocarditis and meningitis). The current, accepted nomenclature for the *Neisseria* species is provided in the Table 1, so the corresponding classifications for generic and specific names allocated in the oldest reports may vary (e.g., ‘*Micrococcus pharyngis siccus*’ in reference [174] refers to *Neisseria sicca*, as stated in the Table 1). Gram-negative diplococci *Moraxella* (*Branhamella*) *catarrhalis* (formely known as *N. catarrhalis*) is a common, essentially harmless inhabitant of the pharynx, but can also behave as an opportunistic pathogen, causing infections mainly in both the upper and lower respiratory tract. Due to its high phenotypic resemblance to the Neisseriae, it was frequently confused with another pharyngeal resident, *Neisseria cinerea* [196]. With this proviso in mind, old case reports of infection with ‘*N. catarrhalis*’ are discussed in the text but are not included in this Table 1 due to its re-classification [197].

## Supplementary Materials

The following are available online at https://www.mdpi.com/2076-0817/9/1/10/s1, Table S1: Glossary for medical terminology, Table S2: Antimicrobial treatments for atypical infections with *Neisseria* species, Table S3: The numbers of *Neisseria* species isolated from different host sites (sources), identified within the pubmlst.org/*Neisseria* database.
