# Supplementary material for: Atypical, Yet Not Infrequent, Infections with Neisseria Species
Source: Pathogens. 2019 Dec 20;9(1):10. doi: 10.3390/pathogens9010010 (PMC7168603; doi:10.3390/pathogens9010010)
Supplement: Supplementary file 1 [file pathogens-09-00010-s001.pdf]

pathogens

MDPI

**Table S1.** Glossary for medical terminology [1,2].

| Disease | Definition | Symptoms (Clinical Presentation) |
|---|---|---|
| Abscess | A swollen area within body tissue (e.g. breast, scalp) containing an accumulation of pus. Usually caused by a bacterial infection. | Redness, pain, warmth, and swelling. The swelling may feel fluid-filled when pressed. The area of redness often extends beyond the swelling. Once the abscesses burst, they usually discharge for several days before gradually healing. |
| Arthritis | Joint inflammation. Arthritis describes around 200 conditions that affect joints, the tissues that surround the joint, and other connective tissue. | Joint pain, tenderness and stiffness, inflammation in and around the joints, restricted movement of the joints, warm red skin over the affected joint, weakness and muscle wasting. |
| Bacteraemia | Transient presence of bacteria in the blood. | Can lead to sepsis. See 'Sepsis'. |
| Botryomycosis | A rare chronic bacterial granulomatous disease that usually involves skin and rarely viscera. | Botryomycosis most commonly affects the skin and presents as subcutaneous nodules, abscesses, large verrucous lesions, ulcers, fistulae, and sinuses with purulent discharge. The lesions generally develop over several months and may drain pus. |
| Bronchiectasis | Long-term condition where the airways of the lungs become abnormally widened, leading to a build-up of excess mucus that can make the lungs more vulnerable to infection. | Persistent cough that usually brings up phlegm (sputum) and breathlessness. The severity of symptoms can vary widely. |
| Bronchitis | Infection of the main airways of the lungs (bronchi), causing them to become irritated and inflamed. | Hacking cough, which may bring up clear, yellow-grey or greenish mucus (phlegm). Sore throat, headache, runny or blocked nose, aches, pains, and tiredness. |
| Cavitary lung lesion | A lung cavity is defined radiographically as a lucent area contained within a consolidation, mass, or nodule. Cavities usually are accompanied by thick walls, greater than 4 mm. These should be differentiated from cysts, which are not surrounded by consolidation, mass, or nodule, and are accompanied by a thinner wall. The differential diagnosis of a cavitary lung lesion is broad and can be delineated into | Many different diseases present as cavitary pulmonary nodules. The spectrum of diseases ranges from acute to chronic infections, chronic systemic diseases, and malignancies. A benign infection may cause hemoptysis when affecting a nearby vessel. Benign diseases may also cause fatigue and weight loss similar to malignancies. Acute onset of fever is usually helpful to distinguish benign disorders from malignancies, but a pulmonary cancer may present with a superinfection secondary to the tumor. |

| | | |
|---|---|---|
| | categories of infectious and non-infectious etiologies. | |
| Cellulitis | Common and sometimes painful inflammation owing to bacterial infection of the deep subcutaneous layer. | It may first appear as a red, swollen area that feels hot and tender to the touch. The redness and swelling can spread quickly. It most often affects the skin of the lower legs, although the infection can occur anywhere on the body or face. |
| Cervicitis | Cervicitis is an inflammation of the cervix, the lower, narrow end of the uterus that opens into the vagina. | Most often, cervicitis causes no signs and symptoms. If symptomatic, symptoms may include large amounts of unusual vaginal discharge, frequent, painful urination, pain during intercourse, bleeding between menstrual periods and vaginal bleeding after intercourse, not associated with a menstrual period. |
| Conjunctivitis | Eye condition caused by infection or allergies. Conjunctivitis is also known as red- or pink eye. | It usually affects both eyes and makes them bloodshot, burn or feel gritty, produce pus that sticks to lashes, itch, and water. |
| Cutaneous (bacterial) infection | This occurs when bacteria enter the body through a break in the skin, such as a cut or a scratch. | Common symptoms include redness of the skin and a rash. Oher symptoms may be itching, pain, and tenderness. Formation of pus-filled blisters. Skin infections can spread beyond the skin and into the bloodstream. |
| Cystitis | Inflammation of the bladder, usually caused by a bladder infection. | The main symptoms of cystitis include pain, burning or stinging on urination, urge to urinate more often than normal, urine that's dark, cloudy or strong smelling, pain low down in your abdomen, feeling generally unwell, achy, sick and tired. Possible symptoms in young children may also include high temperature (fever) of 38 °C or above, weakness or irritability, reduced appetite and vomiting. |
| Disseminated Gonococcal Infection (DGI) | DGI is a complication of untreated gonorrhea and results from bacteremia of *Neisseria* gonorrhea. Also known as "arthritis-dermatitis syndrome". | Symptoms and signs include fever or chills, arthritis or arthralgias (joint pain), tenosynovitis, multiple skin lesions (skin rash with pink or red spots that become filled with pus) and feeling ill or generally unwell (malaise). |
| Empyema | Pockets of pus that have collected inside a body cavity. The term empyema is most commonly used to refer to pus-filled pockets that develop in the pleural space. | Fever and night sweats, lack of energy, difficulty breathing, weight loss, chest pain, cough and coughing up mucus containing pus. |

| | | |
|---|---|---|
| Endocarditis | Rare and potentially fatal infection of the inner lining of the heart (the endocardium). Commonly caused by bacteria entering the blood and travelling to the heart. | Symptoms of endocarditis are similar to flu and include high temperature, chills, headache, joint and muscle pain. Without treatment, damage of the heart valves may disrupt the normal flow of blood through the heart, triggering a range of life-threatening complications, such as heart failure and stroke. |
| Endometritis | Inflammation of the inner lining of the uterus (endometrium). It is also part of spectrum of diseases that make up pelvic inflammatory disease. | Fever, lower abdominal pain, and abnormal vaginal bleeding or discharge. |
| Endophthalmitis | Inflammation of the interior of the eye. | Eye pain that becomes worse after surgery or injury to the eye, decrease or loss of vision, red eyes, pus from the eye and swollen eyelids. Complications may lead to panophthalmitis (progression to involve all the coats of the eye), corneal ulcer and/or orbital cellulitis. |
| Fistula | Abnormal pathway between two anatomic spaces or a pathway that leads from an internal cavity or organ to the surface of the body (e.g. an oral fistula is a pathological communication between the oral cavity and maxillary sinus) | The typical indication of an oral or dental fistula or sinus tract is a bump that develops on the gum tissue or gingiva, where it is called a ‘gum boil’, or in proximity to an abscessed tooth. The bump might alternatively appear and disappear, and is a sign that infection exists and the body is using the fistula to drain it. Since draining releases the pressure of the abscess, the fistula itself is often not painful, although many patients report an unpleasant taste. |
| Gonorrhea | Sexually transmitted infection (STI) caused by *Neisseria gonorrhoeae* (gonococcus). | Typical symptoms of gonorrhea include a thick green or yellow discharge from the vagina or penis, pain on urination and in women, bleeding between periods. However, around 1 in 10 infected men and almost half of infected women do not experience any symptoms. |
| Intrauterine infection | Infection within the womb, which, in the context of pregnancy, usually means infection of the membranes that surround the baby, the umbilical cord, and/or the amniotic fluid. Also known as chorioamnionitis. | Fever, rapid heartbeat, uterine tenderness, discolored, foul-smelling amniotic fluid. |
| Keratoconjunctivitis | Inflammation of the cornea and conjunctiva. | Eye redness, eye pain, excess tears or other discharge from the eye, difficulty opening the eyelid because of pain or irritation, blurred vision, |

| | | |
|---|---|---|
| | | decreased vision, sensitivity to light (photophobia) and a feeling that something is in your eye. |
| Laryngitis | When the voice box or vocal cords in the throat become irritated or swollen. | The main symptoms are a hoarse (croaky) voice, sometimes losing your voice, irritating cough that does not go away, always needing to clear your throat and sore throat. Children can also experience high temperature of 38 °C or above, reduced appetite and/or have difficulty breathing (but this is rare). |
| Mastitis | Condition which causes a woman's breast tissue to become painful and inflamed. | Mastitis usually only affects one breast, and symptoms often develop quickly. Symptoms can include a red, swollen area on the breast that may feel hot and painful to touch, a breast lump or area of hardness on the breast, a burning pain in the breast that may be continuous or may only occur when breastfeeding, and nipple discharge, which may be white or contain streaks of blood. Flu-like symptoms may also develop, such as aches, a high temperature (fever), chills and tiredness. |
| Meningitis | Infection of the protective membranes that surround the brain and spinal cord (meninges). | Symptoms of meningitis develop suddenly and can include high temperature (fever) of 38 °C or above, being sick, headache, rash, stiff neck, a dislike of bright lights (photophobia), drowsiness or unresponsiveness and fits (seizures), positive Kernig and Brudzinski signs. |
| Necrosis | Death of most or all of the cells in an organ or tissue due to disease (e.g. bacterial infection), injury, hyperthermia or failure of the blood supply (as in infarcted tissue). | In its early stages, necrosis typically causes no symptoms, but it gets painful as it progresses. Then, pain may become constant. Specific symptoms vary depending on the affected tissue. |
| Ophthalmia neonatorum | Eye infection that occurs within the first 30 days of life. It is caught during birth by contact with the mother's birth canal that is infected with a sexually-transmitted disease. The infection may be bacterial, chlamydial or viral. Also known as neonatal conjunctivitis or conjunctivitis of the newborn. | Symptoms usually bilateral and include redness, discharge (may be profuse in gonococcal infection) and swelling of lids (may be severe). |
| Osteomyelitis | Infection that most often causes pain in the long bones in the legs, although other bones, such as those in the back or arms, can also be affected. | The symptoms for acute and chronic osteomyelitis are very similar and include fever, irritability, fatigue, nausea, tenderness, redness, and warmth |

| | | |
|---|---|---|
| | | in the area of the infection, swelling around the affected bone and lost range of motion. |
| Otitis | General term for inflammation of the ear. It is subdivided in otitis externa (inflammation of the ear canal), media (inflammation of the portion of the ear internal to the eardrum, and external to the oval window of the inner ear) and interna (labyrinthitis). | Otitis externa often presents with ear pain, swelling of the ear canal, and occasionally decreased hearing. In young children, otitis media may cause crying, irritability, sleeplessness, pulling on the ears, ear pain, headache, neck pain, and a feeling of fullness in the ear. Otitis interna affects the sensory organs for balance and hearing, so inflammation of the inner ear generally causes vertigo. |
| Panophthalmitis | Panophthalmitis is the most extensive ocular involvement in endophthalmitis with inflammation in periocular tissues (inflammation of all coats of the eye: Intraocular and periocular structures). | Severe inflammation of the anterior and posterior segments, frequently accompanied by corneal opacity, scleral abscess, and perforation or rupture. See 'Endophthalmitis'. |
| Parotitis | Inflammation of one or both parotid glands, the major salivary glands located on either side of the face. | Parotitis presents as swelling at the angle of the jaw. Bacterial parotitis presents as a unilateral swelling, where the gland is swollen and tender and usually produces pus at the Stensen's duct. Symptoms include fever, dehydration, chills, fast heartbeat, and breathing if the infection is causing sepsis. |
| Pelvic Inflammatory Disease (PID) | Infection of the female upper genital tract, including the womb, Fallopian tubes and ovaries. | PID often does not cause any obvious symptoms. Most women have mild symptoms that may include one or more of the following: Pain around the pelvis or lower tummy discomfort or pain during sex that's felt deep inside the pelvis, pain on urination, bleeding between periods and after sex, heavy periods, painful periods, unusual vaginal discharge, especially if it is yellow or green, high temperature, and feeling or being sick. |
| Peritonitis | Infection of the inner lining of the abdomen (the peritoneum), which covers internal organs like the kidneys, liver and bowel. | Abdominal tenderness or distention, chills, fever, fluid in the abdomen, not passing any urine, or passing significantly less urine than usual, difficulty passing gas or having a bowel movement and/or vomiting. |
| Petechiae | Small reddish or purplish spot containing blood that appears in skin or mucous membrane as a result of localized hemorrhage. | Specific symptoms vary depending on the affected site. General symptoms may include the spots spreading or getting bigger, rash, bruising, fatigue, and fever. |

| | | |
|---|---|---|
| Pharyngitis | Inflammation of the pharynx (back of the throat). It is most often referred to simply as "sore throat". | Symptoms that accompany pharyngitis vary depending on the underlying condition, including sore, dry, or scratchy throat, difficulty swallowing, and cough. |
| Phlegm | Mixture of saliva and mucus or pus exuded from the respiratory passages, typically as a result of infection or other disease, as in bronchitis or bronchiectasis. Also known as 'sputum' or 'sputa'. | See 'Bronchiectasis' and 'Bronchitis'. |
| Pneumonia | Swelling (inflammation) of the tissue in one or both lungs. It's usually caused by a bacterial infection. | Cough, which may be dry, or produce thick yellow, green, brown or blood-stained mucus (phlegm), difficulty breathing, rapid heartbeat, high temperature, feeling generally unwell, sweating and shivering, loss of appetite, and chest pain. Less common symptoms include coughing up blood (hemoptysis), headaches, fatigue, feeling sick or being sick, wheezing, joint and muscle pain, feeling confused and disorientated, particularly in elderly people. |
| Polyarthralgia | Arthralgia of multiple joints; multiple joint pain. | Joint pain, joint tenderness, stiffness of the joints, swelling of the joints, limited joint movement, weakness and fatigue. |
| Proctitis | Inflammation of the rectum and anus. | A frequent or continuous feeling that you need to have a bowel movement, rectal bleeding, passing mucus through your rectum, rectal pain, pain on the left side of your abdomen, a feeling of fullness in your rectum, diarrhea, and pain with bowel movements. |
| Purulent wound | A wound with heavy or purulent drainage is a localized defect or excavation of the skin or underlying soft tissue that produces large amounts of serous, sanguineous, serosanguineous or purulent discharge. | With most wounds, a small amount of thin, pale colored exudate is normal. In most severe cases, purulent wound drainage is thick with a yellow, green or brown color, with a pungent, strong, foul, fecal, or musty odor. |
| Septicemia or sepsis | Blood poisoning, especially that caused by bacteria or their toxins. | Severe systemic symptoms including sudden high fever with chills, gastrointestinal symptoms including nausea, vomiting and diarrhea, abdominal pain, rigors, hypotension, myalgia, headache, shortness of breath, rapid heart rate (tachycardia), generally feeling unwell, confusion and anxiety. Severe immune responses to bacteremia may result in septic |

| | | |
|---|---|---|
| | | shock and multiple organ dysfunction syndrome, which are potentially fatal. |
| Sinusitis | Swelling or inflammation of the mucous membrane that lines the sinuses (air-filled cavities behind your cheekbones and forehead), usually caused by an infection. Also known as rhinosinusitis. | Pain, swelling, and tenderness around the cheeks, eyes or forehead, blocked nose, reduced sense of smell, green or yellow mucus from the nose, sinus headache, high temperature, toothache, and bad breath. |
| Sputum/sputa | See ‘Phlegm’. | See ‘Bronchiectasis’ and ‘Bronchitis’. |
| Stomatitis | Inflammation affecting the mucous membranes of the mouth and lips, with or without oral ulceration. | Mouth ulcers with a white or yellow layer and red base, usually inside the lips, cheek, or on the tongue, red patches, blisters, swelling, and oral dysesthesia (a burning feeling in the mouth). |
| Systemic Meningococcal Disease (SMD) | Invasive meningococcal infection, which may cause meningitis, septicemia or both. | See ‘Meningitis’ and ‘Septicemia’. |
| Tenosynovitis | Inflammation of the fluid-filled sheath (called the synovium) that surrounds a tendon. Tenosynovitis can be either infectious or non-infectious. Also known as tendon sheath inflammation. | Joint pain, swelling, and stiffness. |
| Tonsillitis | Inflammation of the tonsils (small masses of lymphoid tissue in the throat, one on each side of the root of the tongue). | Sore throat, difficulty swallowing, hoarse or no voice, fever, coughing, headache, feeling sick, earache, feeling tired. Most severe symptoms may include swollen, painful glands in the neck, white pus-filled spots on the tonsils at the back of the throat and bad breath. |
| Ulceration | Formation of a break on the skin or on the surface of an organ. An ulcer forms when the surface cells die and are cast off. | Symptoms vary depending on the location of the ulcer, but generally cause soreness and burning sensation in the affected area. Common examples include tongue, mouth, stomach, and corneal ulceration. More seriously, a penetrating aortic ulcer may cause severe abdominal pain, shortness of breath, pain in the arms or legs, weakness, loss of consciousness, rapid, weak pulse, heavy sweating, anxiety, pale skin, and nausea. |
| Urethritis | Inflammation of the urethra, the tube that carries urine from the bladder out of the body. It is usually caused by an infection. The term ‘non- | Males with urethritis may experience burning sensation while urinating, itching or burning near the opening of the penis, presence of blood in the semen or urine and/or discharge from the penis. Symptoms of urethritis in |

| | | |
|---|---|---|
| | gonococcal urethritis' is used when the condition is not caused by the sexually transmitted infection gonorrhea. | women include more frequent urge to urinate, discomfort during urination, burning or irritation at the urethral opening and abnormal discharge from the vagina, which may also be present along with the urinary symptoms. |
| Vaginitis | Inflammation of the vagina. | Discharge, itching, burning, and possibly pain. |

Table S1 Legend. Clinical definitions and presentations were sourced mainly from references [1] and [2] with little or no alteration to ensure accuracy.

## References

1. The NHS website. *https://www.nhs.uk/*.
2. Kumar, P.; Clark, M.L. Clinical Medicine. Ninth Edition. *Saunders* **2016**, *ISBN: 9780702065989*.

*pathogens*

MDPI

**Table S2.** Antimicrobial treatments for atypical infections with *Neisseria* species.

| *Neisseria* species | Disease/Infection | Treatment | Case Report |
|---|---|---|---|
| *N. gonorrhoeae* | Cutaneous manifestations of DGI | 1 g of ceftriaxone intramuscularly daily for a total of seven days as well as one dose of 1 g of azithromycin orally for treatment of concurrent *Chlamydia* infection. | [1] |
| | Conjunctivitis | 2 g of ceftriaxone in combination with azithromycin or doxycycline. | [2] |
| | Hyperacute keratoconjunctivitis and associated asymptomatic urethritis | Parenteral ceftriaxone and every hour moxifloxacin drops. Weeks of therapy, including full-thickness corneal transplant. | [3] |
| | Conjunctivitis with or without associated preseptal cellulitis | Some patients received topical antibiotics only (for gonococcal or chlamydial conjunctivitis): ofloxacin drops 6 hourly; fucidic acid drops 12 hourly + ofloxacin drops 4 hourly; or erythromycin drops 6 hourly + ofloxacin drops 6 hourly. Some of these also received an initial systemic treatment with oral ciprofloxacin 500 mg 12 hourly + oral azithromycin 1 g stat; or intravenous ceftriaxone 1 g stat + oral azithromycin 1 g stat. After swab results, the treatment to some of these patients changed to oral cefaclor 250 mg 8 hourly + gentamicin drops 6 hourly; oral cefixime 400 mg 12 hourly + oral azithromycin 1 g stat; intravenous ceftriaxone 1 g stat; or oral ciprofloxacin 500 mg 8 hourly + oral azithromycin 1 g stat.<br>Other patients were initially treated for associated presumed preseptal cellulitis with chloramphenicol drops 6 hourly, some of whom also received an initial systemic treatment with oral co-amoxiclav 625 mg 8 hourly; or intravenous flucloxacillin 500 mg 6 hourly + intravenous cefotaxime. After swab results, the treatment to some of these patients changed to intravenous ceftriaxone 1 g stat; or intravenous ceftriaxone 1 g stat + gentamicin drops 6 hourly. | [4] |
| | Pharyngitis | Ceftriaxone in conjunction with azithromycin (in most high-income countries). This dual drug regimen was introduced to limit further development of resistance to ceftriaxone, which is among the last remaining drugs with proven efficacy and safety for the treatment of gonorrhea. Although all genital infections were successfully treated, two pharyngeal infections did not respond to ceftriaxone treatment, probably because drug penetration in the pharynx is poor. An alternative treatment to pharyngeal infections was 3 days of ertapenem administered intravenously. | [5] |

| | | | |
|---|---|---|---|
| | Urethral discharge and dysuria with associated asymptomatic pharyngeal infection | Although the patient's urethral infection was cleared with empirical ceftriaxone/doxycycline treatment, his asymptomatic pharyngeal infection failed treatment despite the relatively high dose of ceftriaxone used (1 g, compared to 250–500 mg frequently used). The patient was then treated with a single 2 g dose of intramuscular spectinomycin. Next, the patient was prescribed 1 g ertapenem intravenously for 3 days. * First reported *ceftriaxone*-resistant, high-level *azithromycin* resistant N. *gonorrhoeae* isolate worldwide. | [6] |
| | Gonorrhea and associated pharyngitis | A single dose of ceftriaxone intramuscularly at a dose of 500 mg plus 1 g of azithromycin orally. At the test of cure on day 15, a urine specimen was negative, but a pharyngeal swab remained positive for N. *gonorrhoeae*. The patient then received one dose of ceftriaxone at a dose of 1 g intramuscularly plus azithromycin at a dose of 2 g orally. | [7] |
| | Nipple piercing wound with associated pharyngeal gonococcal carriage. | 500 mg ceftriaxone by intramuscular injection and azithromycin 1000 mg orally. | [8] |
| | Non-lactational mastitis and breast abscess | Initially prescribed trimethoprim-sulfamethoxazole 160–800 mg twice daily and referred to the breast surgery clinic, but the patient was unable to obtain the antimicrobials. The patient was then prescribed clindamycin 300 mg three times a day and completed a 10-day course of treatment. No improvement, even after surgical aspiration of the affected area. Patient was then prescribed an additional 10 days of clindamycin, which she took for only 1 day. Repeated aspirations of the breast were unsuccessful. Patient was prescribed intramuscular injection of 1 g of ceftriaxone and a prescription for 1 g of azithromycin. The patient was unable to tolerate oral azithromycin and so was referred to the hospital for intravenous (IV) antimicrobials. Patient received 5 days of IV ceftriaxone. Post-aspiration and following clinical improvement, antimicrobials were changed to oral ciprofloxacin 500 mg every 12 h for 10 days. | [9] |
| | Endocarditis | A broad-spectrum antibiotic regimen including benzylpenicillin was begun even though no obvious source of infection was identified. After diagnosis was confirmed, the patient was continued on ceftriaxone for a two-week course of treatment. | [10] |
| *N. meningitidis* | Urethritis | Single 250 mg dose of intramuscular ceftriaxone and single 1 g dose of oral azithromycin. | [11] |
| | | An infection with *N. gonorrhoeae* was assumed and the patient was treated on the spot with ceftriaxone 500 mg intramuscularly and azithromycin 1 g orally (for possible co-infection with *C. trachomatis*). | [12] |

| | | | |
|---|---|---|---|
| | | Presumptive gonococcal infection was treated with 400 mg of cefixime and 1 g of azithromycin orally. Despite misdiagnosis, the patient's dysuria abated 5 days post-treatment (* see female sexual partner with associated cervicitis below). | [13] |
| | *Cervicitis | The female partner was treated with 125 mg intramuscularly ceftriaxone for presumptive oropharyngeal gonococcal infection. Since her male partner tested negative for *C. trachomatis*, no azithromycin was administered. | [13] |
| | Conjunctivitis | Systemic treatment with cefotaxime and ampicillin-sulbactam, together with topical tobramycin and gentamycin. | [14] |
| | | Intensive topical chloramphenicol 0.5% and ofloxacin 0.3% eye drops, combined with 3 doses of intravenous ceftriaxone. By the third day of treatment, the patient was discharged with topical chloramphenicol 0.5% and a course of oral ciprofloxacin. | [15] |
| | Conjunctivitis and sepsis | 7 days of IV ceftriaxone. | [16] |
| | Fulminant endophthalmitis | Because small blisters on her fingertips were noted, infection with herpes simplex virus (HSV) was considered, and therapy with acyclovir was prescribed (15th DOI). Since no improvement was observed, patient was prescribed an oral steroid (up to 1 mg/kg body weight). Then, acyclovir and vancomycin were instilled intravitreally, and intravenous vancomycin for 17 days and ceftazidime for 9 days, followed by cefotaxime for 8 days, was administered. | [17] |
| | Endogenous endophthalmitis and meningitis | Endogenous meningococcal endophthalmitis was treated with topical cefuroxime 5% and dexamethasone 0.1% eyedrops 4 times daily for 20 days. Meningitis was treated with a 4-week course of IV ceftriaxone and oral rifampicin 100 mg twice daily. | [18] |
| | SMD, septicemia, gastrointestinal symptoms | 2 out of 15 patients received IV antibiotics. No treatment informed for the other 13 patients. | [19] |
| | Cellulitis | Appropriate antibiotic therapy (not detailed). | [20] |
| | Necrotizing fasciitis | Initial treatment with benzylpenicillin, 20 million units per day (MU/day) intravenously (IV), and clindamycin, 600 mg IV four times daily. After diagnosis confirmation, the patient was administered ceftriaxone, 2 g IV twice daily. Post-operatively, the patient was prescribed benzylpenicillin, 20 MU/day, later lowered to 12 MU/day for another 7 days. The patient received a MenACWY vaccination to prevent a re-infection in the following 3–5 years. | [21] |
| *N. bacilliformis* | Endocarditis | Initial empiric broad-spectrum antimicrobial coverage with piperacillin–tazobactam, followed by ceftriaxone, gentamicin, rifampin, and doxycycline. | [22] |

| | | | |
|---|---|---|---|
| *N. cinerea* | Neonatal conjunctivitis | The infant received erythromycin eye prophylaxis in the delivery room. The newborn was treated with a 5-day course of tobramycin. | [23] |
| *N. elongata* | Endocarditis | Initial 5-day course of ciprofloxacin, followed by intravenous ceftriaxone 1 g to complete 6 weeks duration of therapy. | [24] |
| *N. flavescens* | Necrotizing pneumonia and empyema | Initial anti-infection therapy with cephalosporin, followed by intravenous injection of cefodizime and methylprednisolone. Finally, one week therapy combining piperacillin-tazobatam and trimethoprim-sulfamethoxazole | [25] |
| *N. mucosa* | Endocarditis | Intravenous ceftriaxone (2 g twice a day) and gentamicin (3 mg/kg/day) were prescribed for 5 days. Gentamicin was then replaced by ciprofloxacin (500 mg, twice a day). Treatment stopped after 6 weeks. A literature review of other 21 similar episodes of *N. mucosa* endocarditis reported alternative antibiotic treatments: ceftriaxone 4 g/day, 6 weeks + gentamicin 3 mg/kg/day, 5 days + ciprofloxacin 1.2 g/day, 6 weeks; ceftriaxone 2 g/day, 6 weeks gentamicin 1 mg/kg three times a day, 4 weeks; ceftriaxone 1 g twice a day followed by ciprofloxacin 200 mg twice a day, 4 weeks; ampicillin 150 mg/kg/day 8 days followed by netilmicin 6 mg/kg/day 8 days; and others (see corresponding reference for more details) | [26] |
| *N. oralis* | Cystitis | The patient was empirically started on a course of trimethoprim, followed by an empiric 14-day course of oral co-amoxiclav. | [27] |
| *N. sicca* | Endocarditis | Initial treatment with rifampin, ceftazidime and a single dose of gentamycin for 3 days. Antibiotic coverage was then narrowed to ceftriaxone for a total of 10 weeks. | [28] |
| | Endocarditis and consequential fatal sepsis | Ciprofloxacin and then doxycycline were initially administered but shown to be ineffective. After diagnosis confirmation, the patient deteriorated rapidly despite adequate antibiotic therapy with amoxicillin/clavulanic acid and gentamicin with consecutive embolic phenomena occurring in the skin, the cerebellum, the conjunctives and the lungs. | [29] |
| *N. sicca/subflava* | Iatrogenic meningitis and associated bacteremia | Intravenous ceftazidime, vancomycin, and dexamethasone were commenced for presumed iatrogenic meningitis. Ceftazidime was then discontinued and intravenous ceftriaxone, 2 g twice daily, was initiated; vancomycin was stopped on confirmation of *N. sicca/subflava* infection. After 7 days, the patient was prescribed a 7-day course of intravenous ceftriaxone followed by a 7-day course of oral ciprofloxacin, 500 mg twice daily. | [30] |
| *N. subflava* | Meningitis | Initial perioperative antimicrobial coverage with cefazolin. After diagnosis confirmation, antibiotic treatment with meropenem was commenced, leading to the gradual resolution of the meningitis. Meropenem was continued for 2 weeks after surgical intervention. | [31] |

| | | | |
|---|---|---|---|
| *N. weaveri* | Peritonitis | Empiric antibiotic treatment was switched to ampicillin/sulbactam, which was administered intraperitoneally 2 g every 12 hours, for a period of 2 weeks. | [32] |

Table S2 Legend. Exemplar antimicrobial treatments of atypical infections with *N. gonorrhoeae*, *N. meningitidis* and commensal *Neisseria* species reported in the last 10 years (2010–2019). Where the antimicrobial dose/concentration is not detailed, it is because no further clinical information was reported. 'stat', immediately. Examples of reported therapies against typical gonococcal and meningococcal infections are not included in this Table. Typical, uncomplicated gonorrhea (gonococcal infection of male and female genitourinary tract) is usually treated empirically with a short course of antibiotics, without testing for antimicrobial susceptibility. The Centers for Disease Control and Prevention (CDC) recommends a single dose of 250 mg of intramuscular ceftriaxone and 1g of oral azithromycin. Cefotaxime, ceftriaxone, and penicillin are preferred as initial therapy in patients with a clinical diagnosis of SMD, although alternative antibiotic therapies to treat typical meningococcal disease are also available [33].

## References

1. Beatrous, S.V.; Grisoli, S.B.; Riahi, R.R.; Matherne, R.J.; Matherne, R.J. Cutaneous manifestations of disseminated gonococcemia. *Dermatol Online J* **2017**, *23*.
2. Belga, S.; Gratrix, J.; Smyczek, P.; Bertholet, L.; Read, R.; Roelofs, K.; Singh, A.E. Gonococcal Conjunctivitis in Adults: Case Report and Retrospective Review of Cases in Alberta, Canada, 2000–2016. *Sex Transm Dis* **2019**, *46*, 47–51, doi:10.1097/OLQ.0000000000000897.
3. Lessing, J.N.; Slingsby, T.J.; Betz, M. Hyperacute Gonococcal Keratoconjunctivitis. *J Gen Intern Med* **2019**, *34*, 477-478, doi:10.1007/s11606-018-4825-8.
4. McAnena, L.; Knowles, S.J.; Curry, A.; Cassidy, L. Prevalence of gonococcal conjunctivitis in adults and neonates. *Eye (Lond)* **2015**, *29*, 875-880, doi:10.1038/eye.2015.57.
5. Regan, D.G.; Hui, B.B.; Wood, J.G.; Fifer, H.; Lahra, M.M.; Whiley, D.M. Treatment for pharyngeal gonorrhoea under threat. *Lancet Infect Dis* **2018**, *18*, 1175–1177, doi:10.1016/S1473–3099(18)30610-8.
6. Eyre, D.W.; Sanderson, N.D.; Lord, E.; Regisford-Reimmer, N.; Chau, K.; Barker, L.; Morgan, M.; Newnham, R.; Golparian, D.; Unemo, M., et al. Gonorrhoea treatment failure caused by a *Neisseria gonorrhoeae* strain with combined ceftriaxone and high-level azithromycin resistance, England, February 2018. *Euro Surveill* **2018**, *23*, doi:10.2807/1560–7917.ES.2018.23.27.1800323.
7. Fifer, H.; Natarajan, U.; Jones, L.; Alexander, S.; Hughes, G.; Golparian, D.; Unemo, M. Failure of Dual Antimicrobial Therapy in Treatment of Gonorrhea. *N Engl J Med* **2016**, *374*, 2504–2506, doi:10.1056/NEJMc1512757.
8. Pendle, S.; Barnes, T. *Neisseria gonorrhoeae* isolated from an unexpected site. *Sex Health* **2016**, *13*, 593–594, doi:10.1071/SH16124.
9. Ceniceros, A.; Galen, B.; Madaline, T. Gonococcal breast abscess. *IDCases* **2019**, *18*, e00620, doi:10.1016/j.idcr.2019.e00620.
10. Nie, S.; Wu, Y.; Huang, L.; Pincus, D.; Tang, Y.W.; Lu, X. Gonococcal endocarditis: a case report and literature review. *Eur J Clin Microbiol Infect Dis* **2014**, *33*, 23–27, doi:10.1007/s10096-013-1921-x.
11. Bazan, J.A.; Turner, A.N.; Kirkcaldy, R.D.; Retchless, A.C.; Kretz, C.B.; Briere, E.; Tzeng, Y.L.; Stephens, D.S.; Maierhofer, C.; Del Rio, C., et al. Large Cluster of *Neisseria meningitidis* Urethritis in Columbus, Ohio, 2015. *Clin Infect Dis* **2017**, *65*, 92–99, doi:10.1093/cid/cix215.
12. Genders, R.E.; Spitaels, D.; Jansen, C.L.; van den Akker, T.W.; Quint, K.D. A misleading urethral smear with polymorphonuclear leucocytes and intracellular diplococci; case report of urethritis caused by *Neisseria meningitidis*. *J Med Microbiol* **2013**, *62*, 1905-1906, doi:10.1099/jmm.0.059378-0.
13. Katz, A.R.; Chasnoff, R.; Komeya, A.; Lee, M.V. *Neisseria meningitidis* urethritis: a case report highlighting clinical similarities to and epidemiological differences from gonococcal urethritis. *Sex Transm Dis* **2011**, *38*, 439–441, doi:10.1097/OLQ.0b013e3181ffa7dc.

14. Unal Yilmaz, G.; Alkan, M.; Vatansever Ozbek, U.; Tugrul, H.M. [Healthcare-associated *Neisseria meningitidis* W135 conjunctivitis]. *Mikrobiyol Bul* **2013**, *47*, 722–726.
15. Dryden, A.W.; Rana, M.; Pandey, P. Primary meningococcal conjunctivitis: an unusual case of transmission by saliva. *Digit J Ophthalmol* **2016**, *22*, 25–27, doi:10.5693/djo.02.2015.09.001.
16. Chacon-Cruz, E.; Alvelais-Palacios, J.A.; Rodriguez-Valencia, J.A.; Lopatynsky-Reyes, E.Z.; Volker-Soberanes, M.L.; Rivas-Landeros, R.M. Meningococcal Neonatal Purulent Conjunctivitis/Sepsis and Asymptomatic Carriage of *N. meningitidis* in Mother's Vagina and Both Parents' Nasopharynx. *Case Rep Infect Dis* **2017**, *2017*, 6132857, doi:10.1155/2017/6132857.
17. Kallinich, T.; von Bernuth, H.; Kuhns, M.; Elias, J.; Bertelmann, E.; Pleyer, U. Fulminant Endophthalmitis in a Child Caused by *Neisseria meningitidis* Serogroup C Detected by Specific DNA. *J Pediatric Infect Dis Soc* **2016**, *5*, e13-16, doi:10.1093/jpids/piw012.
18. Yusuf, I.H.; Sipkova, Z.; Patel, S.; Benjamin, L. *Neisseria meningitidis* endogenous endophthalmitis with meningitis in an immunocompetent child. *Ocul Immunol Inflamm* **2014**, *22*, 398–402, doi:10.3109/09273948.2013.854392.
19. Campbell, H.; Parikh, S.R.; Borrow, R.; Kaczmarski, E.; Ramsay, M.E.; Ladhani, S.N. Presentation with gastrointestinal symptoms and high case fatality associated with group W meningococcal disease (MenW) in teenagers, England, July 2015 to January 2016. *Euro Surveill* **2016**, *21*, doi:10.2807/1560–7917.ES.2016.21.12.30175.
20. Carrascosa, M.F.; Casuso-Saenz, E.; Salcines-Caviedes, J.R. *Neisseria meningitidis* cellulitis. *Int J Infect Dis* **2012**, *16*, e760, doi:10.1016/j.ijid.2012.04.014.
21. Russcher, A.; Fanoy, E.; van Olden, G.D.J.; Graafland, A.D.; van der Ende, A.; Knol, M.J. Necrotising fasciitis as atypical presentation of infection with emerging *Neisseria meningitidis* serogroup W (MenW) clonal complex 11, the Netherlands, March 2017. *Euro Surveill* **2017**, *22*, doi:10.2807/1560–7917.ES.2017.22.23.30549.
22. Abandeh, F.I.; Balada-Llasat, J.M.; Pancholi, P.; Risaliti, C.M.; Maher, W.E.; Bazan, J.A. A rare case of *Neisseria bacilliformis* native valve endocarditis. *Diagn Microbiol Infect Dis* **2012**, *73*, 378–379, doi:10.1016/j.diagmicrobio.2012.05.006.
23. Fiorito, T.M.; Noor, A.; Silletti, R.; Krilov, L.R. Neonatal Conjunctivitis Caused by *Neisseria cinerea*: A Case of Mistaken Identity. *J Pediatric Infect Dis Soc* **2018**, 10.1093/jpids/piy116, doi:10.1093/jpids/piy116.
24. Samannodi, M.; Vakkalanka, S.; Zhao, A.; Hocko, M. *Neisseria elongata* endocarditis of a native aortic valve. *BMJ Case Rep* **2016**, *2016*, doi:10.1136/bcr-2015-213311.
25. Huang, L.; Ma, L.; Fan, K.; Li, Y.; Xie, L.; Xia, W.; Gu, B.; Liu, G. Necrotizing pneumonia and empyema caused by *Neisseria flavescens* infection. *J Thorac Dis* **2014**, *6*, 553–557, doi:10.3978/j.issn.2072–1439.2014.02.16.
26. Pilmis, B.; Lefort, A.; Lecuit, M.; Join-Lambert, O.; Nassif, X.; Lortholary, O.; Charlier, C. Endocarditis due to *Neisseria mucosa*: case report and review of 21 cases: a rare and severe cause of endocarditis. *J Infect* **2014**, *68*, 601–604, doi:10.1016/j.jinf.2014.02.007.
27. Alamri, Y.; Keene, A.; Pithie, A. Acute Cystitis Caused by Commensal *Neisseria oralis*: A Case Report and Review of the Literature. *Infect Disord Drug Targets* **2017**, *17*, 64–66, doi:10.2174/1871526517666161123110613.
28. Kirlew, C.; Wilmot, K.; Salinas, J.L. *Neisseria sicca* Endocarditis Presenting as Multiple Embolic Brain Infarcts. *Open Forum Infect Dis* **2015**, *2*, ofv105, doi:10.1093/ofid/ofv105.
29. Sommerstein, R.; Ramsay, D.; Dubuis, O.; Waser, S.; Aebersold, F.; Vogt, M. Fatal *Neisseria sicca* endocarditis. *Infection* **2013**, *41*, 747-749, doi:10.1007/s15010-012-0393-8.
30. Entesari-Tatafi, D.; Bagherirad, M.; Quan, D.; Athan, E. Iatrogenic meningitis caused by *Neisseria sicca/subflava* after intrathecal contrast injection, Australia. *Emerg Infect Dis* **2014**, *20*, 1023–1025, doi:10.3201/eid2006.131117.
31. Wakui, D.; Nagashima, G.; Otsuka, Y.; Takada, T.; Ueda, T.; Tanaka, Y.; Hashimoto, T. A case of meningitis due to *Neisseria subflava* after ventriculostomy. *J Infect Chemother* **2012**, *18*, 115–118, doi:10.1007/s10156-011-0299-x.

32. Kocyigit, I.; Unal, A.; Sipahioglu, M.; Tokgoz, B.; Oymak, O.; Utas, C. Peritoneal dialysis-related peritonitis due to *Neisseria weaveri*: the first case report. *Perit Dial Int* **2010**, *30*, 116–117, doi:10.3747/pdi.2008.00039.
33. Nadel, S. Treatment of Meningococcal Disease. *J Adolesc Health* **2016**, *59*, S21-28, doi:10.1016/j.jadohealth.2016.04.013.

pathogens

**Table S3.** The numbers of *Neisseria* species isolated from different host sites (sources), identified within the pubmlst.org/*Neisseria* database.

| Source | *Neisseria* Species | No of Isolates in Pubmlst.org/*Neisseria* Database |
|---|---|---|
| Blood | *meningitidis* | 7038 |
| | *gonorrhoeae* | 10 |
| | *subflava* | 5 |
| | *Neisseria sp.* | 3 |
| | *bacilliformis* | 2 |
| | *polysaccharea* | 2 |
| | *sicca* | 1 |
| | *elongata subsp. nitroreducens* | 1 |
| | *mucosa* | 1 |
| | *oralis* | 1 |
| | *perflava* | 1 |
| CSF | *meningitidis* | 8613 |
| | *subflava* | 1 |
| Joint fluid | *meningitidis* | 52 |
| | *gonorrhoeae* | 6 |
| Urethral swab | *gonorrhoeae* | 507 |
| | *meningitidis* | 57 |
| | *lactamica* | 1 |
| Throat swab | *meningitidis* | 8229 |
| | *lactamica* | 730 |
| | *gonorrhoeae* | 53 |
| | *Neisseria sp.* | 14 |
| | *polysaccharea* | 9 |
| | *subflava* | 7 |
| | *cinerea* | 5 |
| | *elongata subspp. elongata* | 5 |
| | *mucosa* | 5 |
| | *macacae* | 1 |
| | *bergei* | 1 |
| Sputum | *meningitidis* | 253 |
| | *lactamica* | 4 |
| | *gonorrhoeae* | 3 |
| | *Neisseria sp.* | 2 |
| | *cinerea* | 2 |
| | *polysaccharea* | 1 |
| | *subflava* | 1 |
| Rectal swab | *gonorrhoeae* | 69 |
| | *meningitidis* | 32 |
| Other * | *meningitidis* | 283 |
| | *gonorrhoeae* | 56 |
| | *Neisseria sp.* | 8 |

| | | |
|---|---|---|
| | *subflava* | 7 |
| | *oralis* | 5 |
| | *cinerea* | 5 |
| | *mucosa* | 4 |
| | *polysaccharea* | 4 |
| | *lactamica* | 3 |
| | *bacilliformis* | 2 |
| | *weaveri* | 1 |
| | *wadsworthii* | 1 |
| | *sicca* | 1 |
| | *musculi* | 1 |
| | *flavescens* | 1 |
| | *dentiae* | 1 |
| | *canis* | 1 |
| | *animalis* | 1 |

Table S3 Legend. Isolation of *Neisseria* species from different host sites does not necessarily imply infection or disease, as in the case of normal carriage of commensal *Neisseria* species in the pharynx ('Throat swabs'). * Undefined by source, but diseases are classified as invasive/septicaemia/meningitis, uncomplicated gonorrhoea, or carrier state. PubMLST.org/*Neisseria* database accessed December, 2019.
